# Liquid biopsy on the horizon in immunotherapy of non-small cell lung cancer: current status, challenges, and perspectives

**DOI:** 10.1038/s41419-023-05757-5

**Published:** 2023-03-31

**Authors:** Ying Yang, Hongyang Liu, Youming Chen, Nan Xiao, Zhaoyang Zheng, Hongchun Liu, Junhu Wan

**Affiliations:** 1 Department of Clinical Laboratory, The First Affiliated Hospital of Zhengzhou University, Zhengzhou, Henan China; 2 Department of Obstetrics and Gynecology, The Third Affiliated Hospital of Zhengzhou University, Zhengzhou, Henan China; 3 Department of Clinical Laboratory, The Second Affiliated Hospital of Henan University of Traditional Chinese Medicine, Zhengzhou, Henan China

**Keywords:** Lung cancer, Prognostic markers

## Abstract

Non-small cell lung cancer (NSCLC) is one of the most threatening malignancies to human health and life. In most cases, patients with NSCLC are already at an advanced stage when they are diagnosed. In recent years, lung cancer has made great progress in precision therapy, but the efficacy of immunotherapy is unstable, and its response rate varies from patient to patient. Several biomarkers have been proposed to predict the outcomes of immunotherapy, such as programmed cell death-ligand 1 (PD-L1) expression and tumor mutational burden (TMB). Nevertheless, the detection assays are invasive and demanding on tumor tissue. To effectively predict the outcomes of immunotherapy, novel biomarkers are needed to improve the performance of conventional biomarkers. Liquid biopsy is to capture and detect circulating tumor cells (CTCs), circulating tumor DNA (ctDNA) and exosomes in body fluids, such as blood, saliva, urine, pleural fluid and cerebrospinal fluid as samples from patients, so as to make analysis and diagnosis of cancer and other diseases. The application of liquid biopsy provides a new possible solution, as it has several advantages such as non-invasive, real-time dynamic monitoring, and overcoming tumor heterogeneity. Liquid biopsy has shown predictive value in immunotherapy, significantly improving the precision treatment of lung cancer patients. Herein, we review the application of liquid biopsy in predicting the outcomes of immunotherapy in NSCLC patients, and discuss the challenges and future directions in this field.

## Facts

- The field of tumor immunotherapy has been developing rapidly. Represented by immune checkpoint inhibitors, immunotherapy has good efficacy for some patients with non-small cell lung cancer.
- As a non-invasive technique, liquid biopsy can provide valuable diagnostic, prognostic and therapeutic response information by detecting patients’ blood and other body fluids, and has promising clinical applications.
- Multiple biomarkers from liquid biopsy for prognostic evaluation of immunotherapy in patients with non-small cell lung cancer are under extensive investigation.

## Open questions

- Is there a biomarker that can accurately screen the population for immunotherapy benefit and predict immune-related adverse events?
- How can more liquid biomarkers be translated from preclinical studies to clinical applications?
- What are the optimal strategies and urgent challenges for liquid biopsy to monitor lung cancer treatment in the near future?

## Introduction

Lung cancer is one of the most deadly and common types of cancer in the world [1]. Based on histopathology, lung cancer is often clinically classified into non-small cell lung cancer (NSCLC) and small cell lung cancer (SCLC). NSCLC is the major pathological subtype of lung cancer, accounting for ~85% of all lung cancers. Standard therapy for lung cancer relies on the stage of this disease. Typical treatment options are surgical excision combined with a chemotherapy and/or radiotherapy treatment. Four to six cycles of cisplatin or carboplatin are the standard chemotherapy for patients with advanced NSCLC [2]. Over the last two decades, the treatment pattern and prognosis of advanced NSCLC patients have undergone tremendous changes. Firstly, the presentation of targeted therapies against oncogene-addicted tumors has improved patient survival results. Nevertheless, only 15% of advanced NSCLC patients have EGFR, ALK, BRAF or ROS1 gene alterations [3], and secondary drug resistance emerges in many patients during therapy [4, 5]. In addition to targeted therapies, the development of immunotherapy represented by immune checkpoint inhibitors (ICIs) has been changing rapidly.

The opening of the immunotherapy era brings new hope to lung cancer patients. ICIs targeting programmed cell death protein 1 (PD1) or programmed cell death ligand 1 (PD-L1) have been determined to be the standard of care for NSCLC. ICI is a breakthrough immunotherapy with excellent long-term survival rates in patients who achieve a complete response. Either immunotherapy as monotherapy (first and second line, in conditions with ≥50% PD-L1 expression) or immunotherapy combined with chemotherapy (first line, in patients with <50% PD-L1 expression), the 5-year overall survival (OS) rate was 20% in unselected patients and up to 40–50% in patients with high PD-L1 expression [6–8]. Unfortunately, there is only a small proportion of patients benefitting from immunotherapy. Therefore, new biomarkers are needed. Identifying biomarkers that respond optimally to treatment with ICIs in NSCLC patients will not only help to choose patients who will receive benefit from immunotherapy, but also to limit non-effective treatments that may result in adverse reactions in patients.

In this context, there has been an interest in liquid biopsy, a technique that uses human body fluids as a source of specimens for detection and analysis. The clinical use of liquid biopsy has been established in many solid cancers for early screening and monitoring of minimal residual disease (MRD) or acquired treatment resistance [9]. Several cancers are characterized by low concentrations of circulating tumor DNA (ctDNA), such as renal cell carcinoma, brain cancer or prostate cancer, where the application of liquid biopsy is limited [10]. At the same time, colorectal cancer (CRC), breast cancer (BC) and melanoma have shown that liquid biopsy can be used in clinical routine [11]. The most widely researched components of liquid biopsy in the field of NSCLC are circulating tumor cells (CTCs), circulating tumor DNA (ctDNA), and extracellular vesicles (EVs). The advantages of liquid biopsy over tissue biopsy have been widely shown. As a less invasive approach, it allows continuous sampling and surveillance of molecular changes over the entire disease process, thus avoiding the information loss because of tumor heterogeneity, improving the accuracy of efficacy evaluation, and leading to better adjustment of treatment regimens [12].

In this review, we summarize the current status of biomarkers available for monitoring immunotherapy response in patients with NSCLC, highlighting existing liquid biopsy methods and possible new biomarkers. This may contribute to the availability of personalized dynamic biomarkers that can be used in precise oncology settings in the near future.

## Immunotherapy of NSCLC

**Fig. 1:**
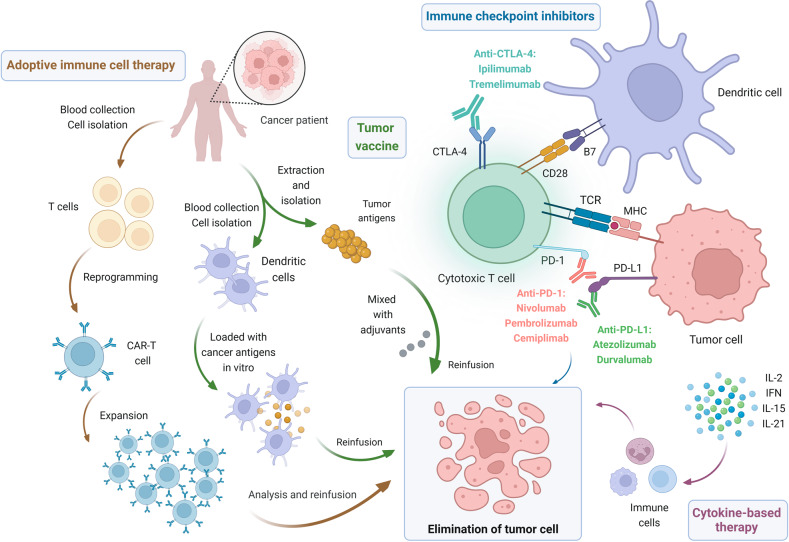
Different immunotherapy approaches. They mainly include immune checkpoint inhibitors, adoptive immune cell therapy, tumor vaccine and cytokine-based therapy. Among the immune checkpoint inhibitors, PD-1/PD-L1 inhibitors block the interaction between PD-1 and PD-L1, preventing the inhibition of anti-tumor activity of cytotoxic T cells and thus eliminating tumor cells. Similarly, CTLA-4 inhibitors enable T cells to proliferate massively and attack tumor cells by binding CTLA-4 molecules. As shown in the figure, the currently approved drugs for NSCLC are *nivolumab*, *pembrolizumab*, *cemiplimab* targeting PD-1; *atezolizumab*, *durvalumab* targeting PD-L1 and *ipilimumab*, *tremelimumab* targeting CTLA-4. Adoptive immune cell therapy is exemplified by chimeric antigen receptor (CAR) T-cell therapy. Technicians isolate and purify T cells from the blood of a tumor patient. A viral vector containing a CAR that recognizes tumor cells and activates T cells is genetically engineered into the T cells, transforming T cells into CAR-T cells. The CAR-T cells are cultured in vitro to expand in large numbers, and then reinfused into the patient’s body, where they can specifically recognize tumor cells and efficiently kill them through immune effect. As for cancer vaccines, one of them involves removing cancer cells from a patient’s tumor, isolating cancer antigens and mixing these antigens with adjuvants that enhance the immune response, and injecting them back into the patient. The immune system can then recognize and attack cancer cells. Another type of cancer vaccine uses dendritic cells. Dendritic cells are removed from the blood and loaded with cancer antigens outside the body. The dendritic cells then take up the cancer antigens and post them on the cell surface. When injected back into the body, these antigen-rich dendritic cells trigger an immune response to the cancer. Finally, cytokine-based therapy suppresses tumors by directly administering exogenous cytokines that effectively activate immune cells in the body or counteract immune suppression.

Tumor immunotherapy is a completely different tumor treatment method from the traditional chemotherapy, radiotherapy and surgery. It applies immunological principles and methods to improve the immunogenicity of tumor cells and their sensitivity to effector cell killing, stimulate and enhance the body’s anti-tumor immune response, so as to achieve the purpose of tumor control and clearance. In recent years, the good news of tumor immunotherapy has continued and has demonstrated powerful anti-tumor effects in the treatment of various tumors such as melanoma, kidney cancer and prostate cancer and other solid tumors [13–15]. The main tumor immunotherapy methods are shown in Fig. 1.

### Immune checkpoint inhibitors (ICIs)

Currently, immunotherapy for lung cancer mostly refers to the application of immune checkpoint inhibitors. Immune checkpoints are a large group of molecules expressed in immune cells, antigen-presenting cells, tumor cells, etc. and play a role in inhibiting or activating the acquired immune system, including PD-1, PD-L1, CTLA-4, LAG3, B7-H3, TIM3, etc. [16]. Cytotoxic T lymphocyte activation associated antigen 4 (CTLA4), the first immune checkpoint, was an important scientific discovery in the 1990s. It is a transmembrane receptor on T cells, also known as CD152, which acts as an immune checkpoint and downregulates the immune response. Researchers have developed monoclonal antibodies against CTLA4 and applied it to the treatment of unresectable metastatic melanoma with promising results. The monoclonal antibody was approved by the Food and Drug Administration (FDA) in 2011 as the first immune checkpoint inhibitor for the treatment of advanced unresectable metastatic melanoma [17]. Since then, the research on immune checkpoints and their inhibitors has entered a period of rapid development. After a decade of development, several new immune checkpoints have been identified, and monoclonal antibody drugs or small molecule inhibitors targeting them have also been developed. Among them, the immune checkpoints that have been studied in depth include programmed death receptor 1 (PD-1) and PD ligand-1 (PD-L1).

**Fig. 2:**
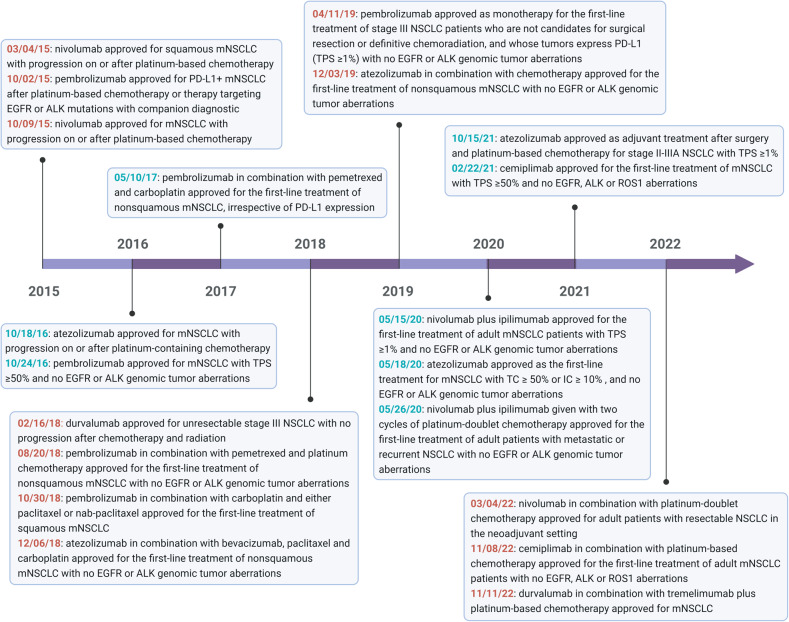
A timeline illustrates the immune checkpoint inhibitors (ICIs) approved by the Food and Drug Administration for NSCLC to date. mNSCLC metastatic NSCLC, TPS tumor cell proportion score, TC tumor cell, IC immune cell.

Programmed death receptor 1 (PD-1) is an immune checkpoint receptor expressed by activated T cells and an important immunosuppressive molecule. Tumor cells can express its ligand PD-L1. When PD-1 and PD-L1 are combined, they deliver negative regulatory signals to T cells, resulting in the inability of T cells to recognize tumor cells and the immune escape of tumor cells [18]. PD-1/PD-L1 inhibitors can specifically bind to PD-L1 on tumor cells to inhibit its expression, thereby restoring recognition of tumor cells by functionally suppressed T cells and achieving anti-cancer effects. Blocking the PD-1/PD-L1 pathway has become the focus of cancer immunotherapy. In 2015, results from the clinical trials CheckMate 017 and CheckMate 057 revealed that NSCLC patients treated with the anti-PD-1 monoclonal antibody nivolumab had a median OS extension of ~3 months compared to treatment with doxorubicin (median OS of 9.2 months and 6.0 months, respectively) [19]. Subsequently, nivolumab became the first checkpoint inhibitor authorized by FDA for 2nd-line treatment of NSCLC. In 2016, Pembrolizumab (anti-PD-1) was also approved by the FDA for first- or second-line monotherapy in NSCLC patients [20–22]. As clinical studies in the field of tumor immunotherapy are rapidly developing, more and more immune checkpoint inhibitors are being approved for NSCLC therapy (Fig. 2).

Combining anti-PD-1/PD-L1 drugs with chemotherapy or other ICIs (such as those targeting CTLA-4) can improve the prognosis of patients with NSCLC. In the randomized, phase III KEYNOTE 407 trial, compared with chemotherapy alone, the addition of pembrolizumab to chemotherapy with carboplatin plus paclitaxel or nab-paclitaxel significantly prolonged OS and progression-free survival (PFS) in patients with metastatic squamous NSCLC [23]. Another study combining nivolumab (anti-PD-1) and ipilimumab (anti-CTLA-4) with two cycles of platinum-based chemotherapy also had better results in comparison to first-line chemotherapy [24]. Lately, several emerging immune checkpoints with possible therapeutic effects have been confirmed, and the most promising seems to be LAG-3, TIM-3, B7-H3 and TIGIT [25]. Among them, LAG-3 has been acclaimed as a new generation of tumor immunotherapy target after PD-1, with great application prospects. A breakthrough in cancer immunotherapy has been made with this technique, and more and more ICIs are expected to be approved for lung cancer treatment by the FDA in the near future. With the important breakthrough of this method in cancer immunotherapy, it is believed that more and more ICI will be approved by FDA for the treatment of lung cancer patients.

### Adoptive immune cell therapy

Adoptive immune cell therapy, also known as cellular immunotherapy, works by directly isolating autologous or allogeneic immune cells and transfusing them back into the patient’s body through in vitro modification and expansion to kill tumor cells [26]. At present, adoptive immune cell therapy mainly includes chimeric antigen receptor (CAR) T-cell therapy, natural killer (NK) cell therapy and γδ T-cell therapy, etc.

Kymriah (tisagenlecleucel, CTL019), developed by Novartis, was approved for marketing by the FDA in August 2017 for the treatment of pediatric and young patients (≤25 years old) with relapsed/refractory B-cell acute lymphoblastic leukemia (R/R B-ALL). It is the first approved CAR-T therapy in the world. 2022 European Hematology Association (EHA) Congress announced 5-year long-term follow-up results showing an overall 5-year survival rate of 55% in 79 patients receiving Kymriah therapy in the final ELIANA trial analysis. While currently being studied with relative success in hematologic malignancies, CAR-T therapies have struggled to break through in lung cancer. Selecting appropriate targets and chimeric antigen receptors and overcoming immunity of the tumor microenvironment are two directions that CAR-T needs to solve in solid tumors [27]. Some researchers have constructed CAR-T cells targeting PD-L1, which proved that PD-L1-CAR-T cells had anti-tumor activity in vitro, and the xenograft tumors of NSCLC with high expression of PD-L1 in mice also achieved prolonged remission [28]. This finding provides preclinical evidence to support the targeting of PD-L1 by CAR-T cells for the treatment of NSCLC and other solid malignancies.

### Tumor vaccine

Tumor vaccine is to introduce tumor antigens into patients in various forms (such as tumor cells, tumor related proteins or polypeptides, genes expressing tumor antigens, etc.), overcome the immunosuppressive state caused by tumors, enhance immunogenicity, activate the patient’s own immune system, induce cellular and humoral immune responses, so as to control or eliminate tumors. Scientists at the University of California, Los Angeles (UCLA) have discovered a dendritic cell vaccine, CCL21-dendritic vaccine [29], which amplifies the immune system response to NSCLC. The CCL21-dendritic vaccine was directly applied to the tumors of 16 patients with NSCLC, and at day 56, 25% of the patients had stable disease (meaning their tumors did not increase or decrease in size). In 54% of the patients, CD8 cells infiltrated into the tumor, and the expression of PD-L1 was also significantly increased after vaccination. The preliminary results showed that CCL21-DC vaccine could induce systemic tumor antigen-specific immune response. This study is the first to test the vaccine in humans and has important clinical significance in the field of lung cancer treatment.

### Cytokine-based therapy

Cytokines are polypeptides or glycoproteins with a relative molecular weight of less than 30,000, which provide growth, differentiation, inflammation or anti-inflammatory signals for different cell types, and can effectively activate tumor immune cells or counteract immunosuppression, thereby inhibiting tumors. Immunotherapy based on several important cytokines such as IL-2, IL-21 and GM-CSF has been widely studied in tumor immunotherapy [30–32].

## Biomarkers in NSCLC immunotherapy

Despite the impressive results of immunotherapy for tumors in recent years, a considerable proportion of patients do not benefit from immunotherapy and the exact mechanisms involved remain unclear. In addition, ICIs that increase the tumor-killing activity of the host immune system may also lead to the development of immune-related adverse events (irAEs), and these reactions may be life-threatening in some cases [33]. Therefore, accurate biomarkers are essential for screening populations for immunotherapy benefit and for predicting irAEs. Current immunotherapy guidelines are based primarily on patients’ PD-L1 levels on the primary tumor or recent tumor mutation burden (TMB) levels to determine whether they are eligible for PD-1/PD-L1 inhibitor therapy [34]. However, these biomarkers are not optimally accurate. There is still a need to discover novel predictive immunotherapy biomarkers.

### PD-L1 expression

PD-L1 expression is the most recognized predictor of response to anti-PD1/PD-L1 ICIs and is used to guide therapeutic decisions in metastatic NSCLC. In patients with advanced NSCLC and PD-L1 expression on at least 50% of tumor cells, pembrolizumab treatment showed significantly longer PFS and OS compared to chemotherapy [21]. It was also demonstrated in the Keynote-042 trial, and furthermore, the group of patients with ≥50% PD-L1-positive cells in the primary tumor showed a better prognosis in comparison with the group with PD-L1-positive cells between 1 to 49% [35]. Therefore, higher expression of PD-L1 predicts a better ICI treatment response. Based on this correlation, PD-L1 expression assessment before treatment can help identify patients who can benefit from immunotherapy. As for the detection of PD-L1 expression, different methods rely on immunohistochemical staining and the proportion of PD-L1 cells positivity [36]. The ESMO (European Society of Medical Oncology) and NCCN (National Comprehensive Cancer Network) guidelines propose that anti-PD-1/PD-L1 agents are recommended as first-line monotherapy only when the expression of PD-L1 in NSCLC patients is ≥50% and there are no other operable molecular markers. In NSCLC patients with PD-L1 expression of 1–49%, ICI therapy can be used as second-line monotherapy or in combination with chemotherapy as first-line therapy [34]. For patients with PD-L1 expression <1%, immunotherapy is not recommended, but can be considered in the absence of other treatment options.

In recent years, immunotherapy has evolved from late treatment to early treatment (neoadjuvant/adjuvant) field. The predictive role of PD-L1 in the non-metastatic setting is also emerging. A study on stage III unresectable NSCLC showed that PD-L1-positive patients showed a better prognosis than PD-L1-negative patients after treatment with durvalumab [37]. However, there are also studies with different results, which concluded that PD-L1 expression was not associated with patient prognosis [38]. In patients with stage IB-IIIA NSCLC treated with atezolizumab after surgery and adjuvant chemotherapy, disease-free survival (DFS) improved in the set of patients with PD-L1 ≥ 1%, while the set with PD-L1 ≥ 50% benefited the most [39].

Despite this, PD-L1 expression is far from being a perfect biomarker. First, the screening of the immunotherapy beneficiary population using PD-L1 expression is not entirely accurate. Some patients with high PD-L1 expression do not benefit from ICI therapy in clinic [40, 41], while patients who do not express PD-L1 may respond to this therapy [42]. In addition, PD-L1 can be expressed in both tumor cells and immune cells and there is intratumoral and spatiotemporal heterogeneity, and its detection is influenced by subjective interpretation and lack of standardization across different platforms.

### Tumor mutational burden (TMB)

In general, tumor mutational burden (TMB) refers to the amount of somatic nonsynonymous mutations or all mutations per megabase in gene regions detected in tumor samples (tumor tissue or peripheral blood) [43]. These mutations can lead to neoantigen formation, contribute to the immunogenicity of the tumor [44, 45], and improve the possibility of patient response to ICI treatment [46]. In NSCLC patients with a TMB equivalent to or greater than 10 mut/Mb, the combination of ipilimumab and nivolumab compared to chemotherapy was reported to have a better overall response (OR) and PFS, regardless of PD-L1 expression [47, 48]. Recently, pembrolizumab has been approved by FDA as an ICI for patients with any tumor type. These tumor types, particularly solid cancers, demonstrated high TMB in the KEYNOTE-158 trial [49].

As a marker of therapeutic effects, TMB also has shortcomings. Firstly, similar to PD-L1, the detection approaches for TMB are still not uniform. Whole exome sequencing (WES) was the original proposed strategy to measure TMB [50], but high cost and time-consuming nature limited its wide application. A number of smaller next generation sequencing (NGS) panels have now been developed and verified for calculations of TMB, but only two validation panels have received FDA approval so far: the Foundation One CDx panel and the MSK-IMPACT panel [51–53]. In addition, some studies have shown that not all patients with high TMB respond to ICIs and low TMB can not rule out the likelihood of immunotherapy response [54]. Finally, there is no clearly defined threshold to define a high or low level of TMB [55]. A single TMB threshold is unlikely to be applied uniformly to all analyses, and the FDA’s recommended TMB pan-cancer threshold of ≥10 mut/Mb has been challenged by multiple studies [56–58]. This consensus requires further research to identify TMB as a validated biomarker of response to ICI.

### Other tumor-based markers

ICIs are the current mainstay of immunotherapies. The key lies in the interaction and cross-talk between the immune system and tumor cells. By monitoring biomarkers of the immune microenvironment, such as CD4+ and CD8+ T-cell subsets, neutrophil/lymphocyte ratio, cytokines, etc., we can reflect the game status of the immune system and tumor cells. Tumor microenvironment (TME) is the surrounding environment where tumor cells exist, including surrounding blood vessels, immune cells, fibroblasts, bone marrow-derived inflammatory cells, signal molecules, and the extracellular matrix [59]. It plays an important role in the complex interactions between tumor cells and the immune system. Therefore, all of these participants could potentially be used in biomarker studies.

Several studies have depicted a TME featuring the existence of tumor infiltrating lymphocytes (TILs), the quantity and composition of which can be used as a predictive biomarker of treatment response and prognosis [60]. Intense lymphocytic infiltration predicts longer survival [61, 62], and high density of T lymphocytes (CD4+ and CD8+) in tumor stroma was associated with better outcome [62, 63]. Recently, stratification of TME according to PD-L1 status and the existence of TILs has been suggested, leading to four categories of TME [64]. For NSCLC, type II tumors are the most common, that is, PD-L1-negative tumors with low levels of TIL. Besides, large numbers of infiltrating NK cells and dendritic cells in TME are related to better prognosis after ICIs therapy [65, 66]. Thrombocytes mold TME by producing a powerful immunosuppressive cytokine, transforming growth factor-β1 (TGF-β1), and are therefore also recognized as a biomarker and therapeutic target. Research has demonstrated that in ICIs, high thrombocyte counts in the tumor lead to poorer tumor control [67]. More rigorous clinical studies are needed to further elucidate the role of these cells in lung cancer and their underlying value as biomarkers.

In addition, NSCLC are usually linked to the presence of Tertiary lymphoid structures (TLS), which are ectopic lymphatic structures formed within non-lymphoid tissues that have similar structure and function to lymph nodes [68]. TLS are present in the tumor stroma and margins, with good spatial organization. The existence of TLS is correlated with better response to ICI treatment in various cancer types including NSCLC [69]. Some studies have suggested that the existence of B cells in TLS is related to a good outcome [70]. In particular, a study by Tang et al. showed that in TLS of lung cancer patients with resectable tumors, TLS area and B-cell ratio increased and were associated with longer survival rates [71]. The small molecules in TME are also an available resource for explorable biomarkers, such as interferon-γ (IFN-γ). The potential of TME biomarkers remains to be explored, and the exploitation of TME to develop new therapeutic approach with TME components may indicate the future of cancer immunotherapy.

## Liquid biopsy and the main detection objects

**Fig. 3:**
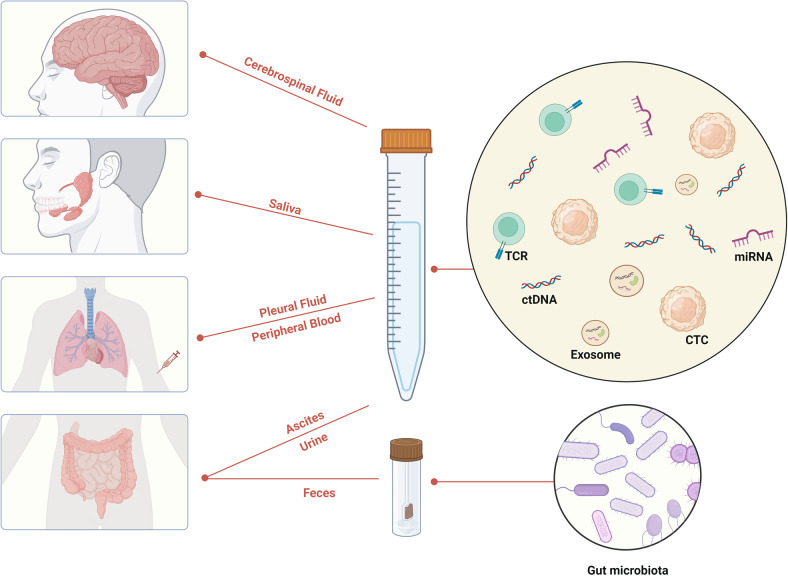
Specimen types and promising biomarkers for liquid biopsy. A variety of body fluids such as cerebrospinal fluid, saliva, pleural fluid, peripheral blood, ascites, and urine are all sources of specimens for liquid biopsy. Blood-based liquid biopsy is currently the most important research direction. The main detection biomarkers are ctDNA, CTCs, exosomes, miRNAs and TCR repertoire, etc. In addition, feces-based biomarkers such as gut microbiota are also promising. ctDNA circulating tumor DNA, CTCs circulating tumor cells, miRNAs microRNAs, TCR T-cell receptor.

As we described above, clinical biomarkers are mostly detected by tumor tissue. However, histopathological biopsy has some defects, such as poor compliance, complex operation, tissue heterogeneity and difficult to detect dynamically, which limits its clinical application. Peripheral blood has the characteristics of tumor homology and simple operation, which is easy to evaluate in real time. It has great potential value as a biomarker carrier. Liquid biopsy is to capture and detect circulating tumor cells (CTC), circulating tumor DNA (ctDNA) and exosomes in body fluids, such as blood, saliva, urine, pleural fluid and cerebrospinal fluid as samples from patients, so as to make analysis and diagnosis of cancer and other diseases (Fig. 3). Compared with traditional tissue biopsy, liquid biopsy has advantages such as non-invasive, real-time dynamic monitoring, and overcoming tumor heterogeneity [72]. The application of liquid biopsy as an emerging detection technology in lung cancer clinics is becoming more and more mature.

### Circulating tumor DNA

Apoptosis or necrosis during normal physiological activity in healthy individuals generates small double-stranded fragments of free DNA that are released into the peripheral blood, called circulating free DNA (cfDNA). Most cfDNA is derived from hematopoietic cells [73] and is ~167 bp in length. Since the level of germline DNA from normal cells in the blood remains essentially constant and tumor cells release their DNA into the circulation, abnormally elevated cfDNA levels may be associated with tumor load [74]. Circulating tumor DNA (ctDNA) is the part of cfDNA that originating from tumor cells, which carries tumor-specific genetic characteristics and epigenetic features, such as mutations, insertions, deletions, chromosomal rearrangements, copy number variants and methylation changes, and therefore ctDNA has the potential to become a tumor biomarker. ctDNA is rapidly cleared from the blood with a typical half-life of 15 min to 2 h, thus allowing for real-time monitoring [75]. The commonly used detection methods are NGS and polymerase chain reaction (PCR). However, ctDNA accounts for only 0.01% of cfDNA and the amount is extremely low, so highly sensitive detection methods are needed.

### Circulating tumor cells

Circulating tumor cells (CTCs) are tumor cells that are shed from primary or metastatic foci into the circulation during tumor formation or progression, which can truly reflect tumor load and have the potential to develop into metastatic lesions. CTCs play an important role in tumor metastasis, and their relationship with the clinicopathological characteristics and prognosis of patients has been demonstrated in breast and colorectal cancers [76–78]. In peripheral blood, there may be only 1 CTC for approximately every 10^6^~10^7^ leukocytes [79], so the detection of CTCs requires their isolation and enrichment before analysis. Enrichment and analysis methods based on physical and biological properties of CTCs, such as size, density, deformability, polarity and charge, epithelial cell adhesion molecules, cytokeratin and expressed tumor-associated markers [80]. The main methods for CTCs enrichment are: density-dependent cell separation, cell size-based separation, immunomagnetic bead negative enrichment, magnetophoretic mobility-based separation, enrichment by microfluidic devices and immunomagnetic bead positive enrichment. CTCs are analyzed by reverse transcription PCR, fluorescence in situ hybridization and fluorescence-assisted cell sorting techniques. Additional methods for simultaneous enrichment and analysis of CTCs include fiber-optic array scanning, Cell Search, morphology-based enrichment by membrane filtration, EPISPOT, and Adna Test. There are also emerging technologies such as the separation of tumor cells from leukocytes using acoustically directed microfluidics that help to rapidly and accurately isolate and analyze CTCs from whole blood [81].

### Exosomes

Exosomes are nano-sized, actively secreted extracellular vesicles-like vesicles with membrane structure, containing complex RNA and proteins, with a diameter of 30–150 nm. It was first discovered in 1983 in sheep reticulocytes and named “exosome” by Johnstone in 1987 [82]. In addition to blood, exosomes are found in a variety of other body fluids such as saliva, urine, and cerebrospinal fluid. Exosomes carry a diverse range of biomolecules, including DNA fragments, circular RNA (circRNA), messenger RNA (mRNA), microRNA (miRNA), functional proteins, transcription factors, etc., which can complete the complex information transfer process between cells. Their membrane structure can also express a variety of antigen and antibody molecules, and participate in intercellular information exchange and material exchange, playing a significant role in various physiological and pathological processes, such as intercellular communication, cell migration, differentiation, pro-vascularization, immune response, tumor invasion, etc. It can also be used as a nanocarrier to load genes or drugs to target organs [83]. The methods of exosome extraction include: differential ultracentrifugation, PEG precipitation, acoustic wave separation, density gradient centrifugation, filtration centrifugation, adsorption and immunomagnetic bead method. The methods for exosome identification include: electron microscopy, immunogold labeling technique, real-time fluorescence quantitative polymerase chain reaction, flow cytometry, nanoparticle tracking analysis technique and sequencing.

### Non-coding RNA (ncRNA)

Non-coding RNAs (ncRNAs) include ribosomal RNA (rRNA), small nuclear RNA (snRNA), small nucleolar RNA (snoRNA), miRNA, long non-coding RNA (lncRNA) and circRNA. The common feature of these RNAs is that they can be transcribed from the genome but not translated into proteins, and perform their respective biological functions at the RNA level. Among ncRNAs, miRNAs, lncRNAs and circRNAs are more suitable for application as biomarkers [84]. Common methods for detecting circulating ncRNAs include microarrays, RNA sequencing, Northern blot hybridization and quantitative real-time PCR (qRT-PCR). Circulating ncRNA is more intensively studied in the diagnosis and prognosis of cancer as a liquid biopsy marker with low invasiveness, high specificity and sensitivity.

#### MicroRNAs

MicroRNAs (miRNAs) are a class of endogenous non-coding RNAs with regulatory functions found in eukaryotes, with a size length of about 20–25 nucleotides. They recognize target mRNAs by base complementary pairing and direct silencing complexes to degrade or block translation of target mRNAs depending on the degree of complementarity [85]. MiRNAs play essential roles in a variety of cellular biological processes [86]. Circulating tumor cells, tumor cells at the primary site or that have metastasized, or immune cells infiltrated in the tumor microenvironment may secrete cancer-associated circulating miRNAs [87]. Differential expression of miRNAs in patients with cancer has been described. In addition, miRNAs in microparticles can act as a new type of signaling molecule that mediates intercellular communication from a distance [88].

#### Long non-coding RNAs

Long non-coding RNAs (lncRNAs) consist of 200 or more nucleotides. Previously, lncRNAs have been studied superficially and were thought to have no biological function, existing only as a by-product of the transcription process. With the development of second-generation sequencing technology, researchers have proved that lncRNAs play an essential role in many biological fields such as tumor development, neuroscience and individual development, and are important regulatory molecules of the human genome. Among the many functions of lncRNAs, the main ones related to tumors are maintaining cell growth and proliferation, promoting metastasis and invasion, inducing angiogenesis and inhibiting apoptosis [89, 90]. It has been found that lncRNAs are dysregulated and aberrantly expressed in a number of tumors [91]. Research on lncRNAs in NSCLC has shown that lncRNAs can affect multiple signaling pathways and play a key role in the development and progression of NSCLC [92].

#### Circular RNAs

Circular RNAs (circRNAs) are a category of covalently closed molecules. Recent studies have shown that circRNA molecules are rich in miRNA binding sites and act as miRNA sponge in cells, which in turn unblock the repressive effect of miRNAs on their target genes and increase the expression level of target genes [93, 94]. This mechanism of action is known as the competitive endogenous RNA (ceRNA) mechanism. Structurally, unlike traditional linear RNAs, circRNA molecules have a closed-loop structure and are more stably expressed. Therefore, it is well suited as a biomarker for cancer diagnosis and monitoring [95]. In the field of lung cancer, numerous studies have confirmed that the abnormal expression of circRNAs is associated with tumorigenesis and cancer evolution, demonstrating their potential as biomarkers for diagnosis, prognosis and treatment [96].

## Liquid biopsy in immunotherapy of NSCLC

### ctDNA and immunotherapy of NSCLC

Several studies have evaluated the utility of plasma cfDNA levels as a predictor of clinical benefit in ICIs-treated patients [97, 98]. Reduced cfDNA levels during NSCLC treatment are associated with better outcomes, which supports its potential as a predictive biomarker. But due to the fact that cfDNA production varies both within and between cancer types, the total level of cfDNA cannot be used as informative biomarkers [99]. Quantitative detection of ctDNA in total cfDNA can be achieved by quantitative polymerase chain reaction (QPCR) analysis to detect known mutations. ctDNA can reflect the actual tumor burden and the specific genomic status of disease and therefore might be used as a prognostic and predictive biomarker for ICI therapy.

Total ctDNA level testing may be a cost-effective method to provide initial screening for tumor presence and volume, as total ctDNA levels have been demonstrated to be related to tumor mass size in many types of tumors, including NSCLC [100, 101]. Further, ctDNA has shown potential clinical predictive value for patients with NSCLC in different disease states. Studies of NSCLC patients receiving anti-PD-1/PD-L1 drugs have found that significant decreases in ctDNA levels are linked to immunotherapy clinical response and prolonged survival [102, 103]. Recent data on resectable NSCLC similarly support ctDNA as a neoadjuvant response biomarker. In a phase III study comparing the efficacy of neoadjuvant platinum chemotherapy combined with or without nivolumab in stage IB-IIIA NSCLC, it was emphasized that ctDNA clearance at day 1 cycle 3 post-combination chemotherapy and ICI treatment was correlated with pathologic complete response (pCR) [104]. In NSCLC patients undergoing primary tumor resection, higher plasma ctDNA levels are correlated with a worse OS, and an increase in ctDNA levels could be detected shortly before patient recurrence [105]. In addition, ctDNA analysis can also identify patients who respond to treatment when they have unresectable locally advanced NSCLC, and indicate the need for further treatment. According to Moding et al. [106], among patients with detectable ctDNA after chemotherapy, a significantly better prognosis was seen for patients who received consolidation immune checkpoint inhibition than those who did not, and a decreased ctDNA level early in treatment was associated with better outcomes than an elevated ctDNA level, while the PFS of patients with undetectable ctDNA levels after treatment was significantly higher.

Moreover, ctDNA can also be used as a non-invasive tool to detect point mutations associated with immunotherapy sensitivity. Guibert et al. [107] found poor outcomes in NSCLC patients with mutant PTEN or STK11, while transversion mutations in KRAS and TP53 genes were associated with better responses. Therefore, the researchers proposed an algorithm to classify patients based on an “immune score” and to identify those who would benefit more from treatment with ICIs. “High immune scores” is defined as those patients without mutations in target driver genes (EGFR, ROS1, ALK, and BRAF V600E), PTEN or STK11, but with mutations in TP53 and KRAS [107]. Interestingly, mutations in STK11 are usually correlated with mutations in KRAS [108], and STK11/KRAS co-mutations have been reported to lead to worse survival outcomes in patients treated with ICI [109], in support of a predictive role for this co-mutation.

In addition, ctDNA is also useful as a surrogate indicator for TMB detection. ctDNA sequencing analysis reveals the mutational status of a tumor, as well as its current TMB in peripheral blood (bTMB) [110]. Compared to traditional TMB calculations, bTMB overcomes the limitations of current solid tumor biopsies, and provides a more actual view of tumor characteristics and progression. Some studies have observed higher levels of bTMB in metastatic NSCLC, while lower levels of bTMB are more common in early NSCLC [111]. The role of bTMB as a biomarker for predicting the efficacy of immunotherapy in NSCLC has been demonstrated [112]. Blood-based tumor mutational load levels were measured in an open-label phase 3 randomized clinical trial (MYSTIC) (NCT02453282) (*N* = 1118) [113]. The results showed that bTMB was associated with improved OS, PFS, and overall response rate (ORR) in patients treated with ICI. However, the low amount of available ctDNA limits the use of bTMB. To assess and stratify patients properly, sufficient amounts of high-quality DNA are required. According to Wei et al.’s meta-analysis [114], the limitations of current detection technology and a lack of standardization prevent bTMB from being used as an independent prognostic factor in ICI-treated patients. Other studies have suggested combining bTMB with other parameters to improve the predictive power, such as maximum somatic allele frequency (MSAF) [115], or incorporating parameters to assess tumor heterogeneity [116, 117].

The current challenges in ctDNA applications are mainly the lack of a unified threshold for baseline risk stratification, and multiple detection methods with varying coverage depths, fragment sizes and detection limits [118]. The use of ctDNA in clinical practice is currently limited, but early identification of responders and response assessment remain possible in the near future.

### CTCs and immunotherapy of NSCLC

Specific application scenarios of CTC testing in clinical settings include early screening of high-risk populations, accurate staging of confirmed patients, post-operative monitoring of recurrence and metastasis in early-stage patients, prognosis determination before the start of treatment and efficacy evaluation after each cycle of treatment in advanced patients, real-time analysis of molecular targets (e.g. EGFR, HER2, ALK, etc.) to predict the efficacy of relevant drugs, etc. However, the level of clinical evidence for the above application scenarios varies. At present, a large amount of evidence-based medical evidence has been accumulated regarding the prognostic judgment and efficacy evaluation of advanced patients, which is the most classical clinical application of CTCs. The presence of CTCs has been reported to be an independent adverse prognostic marker in a variety of cancer types [119–121]. In NSCLC patients, circulating CTCs have been shown to be associated with a poor prognosis [122]. As in the study by Mondelo-Macía et al. [123], patients with detectable CTCs using the CellSearch® system had significant shorter PFS and OS than patients with undetectable CTCs. Taminga et al. included 104 advanced NSCLC patients treated with ICI, and one-third of patients had CTC detected in aliquots of 7.5 ml blood with the Cellsearch Circulating Tumor Cell Kit. The existence of baseline CTC (>1) was associated with worse PFS and OS [124]. Similarly, several studies have demonstrated the correlation between CTC count and poorer response as well as survival rates during ICI treatment [97, 125, 126].

In addition to the above, it is known that 45–93% of CTC samples can be evaluated for PD-L1. The majority of studies have explored the relationship between PD-L1-positive CTCs and ICI-treated patients’ prognoses, with mixed results. A study included 24 patients with metastatic NSCLC taking nivolumab, 19 of whom had CTCs with surface expression of PD-L1 at baseline and at 3 months of treatment and had poor outcomes, whereas patients with PD-L1-negative CTCs obtained clinical benefit at 6 months of treatment [127]. Some evidence also indicates that nivolumab-treated patients with high PD-L1 expression at baseline have a poorer outcome [128]. And this study detected PD-L1-positive CTCs in 100% of immunotherapy-naive patients. Furthermore, the dynamic increase in PD-L1+ CTCs during treatment may indicate ICI resistance [129]. In contrast, the results of Ilie et al. [130] showed no significant impact of PD-L1 expression on CTC on the prognosis of immunotherapy. In addition, the results of studies assessing the concordance between the expression of PD-L1 by CTCs and in tissues are also controversial. According to Ilié et al., a 93% concordance was found between tissues and CTCs concerning PD-L1 expression in 106 patients receiving chemotherapy [130], using the platform ISET. Other studies reveal the opposite. In total, 96 NSCLC patients treated in 2nd line using the ISET platform to isolate CTCs were studied by Guibert et al. They found a higher PD-L1 positivity rate for CTCs than for tissues (83% vs. 41%). Therefore, PD-L1 expression in tissue and CTCs was not correlated. Similar results of Guibert and his colleagues were reported by other research groups, yet using different PD-L1 analysis methods and antibodies [128, 129, 131]. Consequently, conflicting results remain in different cohorts due to the lack of standardized methods for detecting PD-L1 expression [132]. There is a need for more research to evaluate standardized methods and PD-L1 antibodies for CTCs detection and analysis, and to further explore the relationship between PD-L1 expression on CTCs and prognosis for immunotherapy.

Furthermore, exploring other markers in CTCs can help identify novel prognostic and diagnostic factors. Compared to ctDNA, CTC has the advantage of having intact cellular morphology and more complete preservation of intracellular material. Therefore, the assays on CTC will be richer and the confidence of the results will be higher. In addition to covering all the assays of ctDNA, CTC assays can also provide transcriptomic, genomic and proteomic information. For example, expression of indoleamine-2,3-dioxygenase (IDO) in CTCs has shown promising results to predict immunotherapy response [133], and IDO+ CTCs are related to shorter PFS and OS. Studies such as high-throughput sequencing of CTCs and single-cell RNA sequencing (scRNA-seq) also contribute to the extension of CTC omics studies to immunotherapy responses of NSCLC in the future. As a whole, CTC count may be the most promising biomarker during ICI treatment. Nevertheless, technical issues as well as controversial study results need to be resolved before CTC can be translated into the clinic.

### Exosomes and immunotherapy of NSCLC

Exosomes, the most common subtypes of extracellular vesicles, are actively involved in cancer development, metastasis and drug resistance, which makes them a biomarker for cancer screening, diagnosis and surveillance [134].

In a study by Del Re et al., PD-L1 mRNA expression was evaluated in circulating exosomes from 18 patients with melanoma or 8 patients with NSCLC, who were treated with pembrolizumab and nivolumab. The data showed that before ICIs treatment, PD-L1 mRNA copies were significantly higher in responders than in non-responders (mean 830.4 vs. 204.0 copies/ml, respectively). After 2 months of ICIs treatment, there was a significant reduction in PD-L1 mRNA copies in responders (mean 242.5 copies/ml), and a significant increase in non-responders (mean 416.0 copies/ml) [135]. This demonstrated the feasibility of detecting plasma exosomal PD-L1 and its relationship with the efficacy of immunotherapy. Another study [136] collected paired tissue specimens and blood specimens from 51 patients with advanced NSCLC to examine the dynamic changes in the expression of PD-L1 in blood after 2 months of treatment with ICIs in patients with advanced NSCLC, including changes in PD-L1 mRNA, exosomal PD-L1 (exoPD-L1) protein, and soluble PD-L1 (sPD-L1). Among 40 patients with advanced NSCLC, PFS, OS, and best of response (BOR) were better for those with PD-L1 mRNA changes ≥2.04. Besides that, in a group of 21 patients with advanced NSCLC, a fold change of exoPD-L1 ≥ 1.86 was identified to be relevant to better outcome and OS, whereas dynamic changes in sPD-L1 did not. This suggests that elevated expression of PD-L1 mRNA and/or exoPD-L1 early in the treatment of ICIs could be used as positive biomarkers of efficacy and prognosis in advanced NSCLC patients. In addition, combining PD-L1 mRNA with exoPD-L1 could allow better screening of patients for the potential benefits of ICIs therapy. However, there are conflicting findings on the concordance of PD-L1 expression in exosomes and tissues. In a study of NSCLC patients by Li et al., there is a correlation between PD-L1 expression in exosomes and disease progression, tumor size, lymphatic status, metastasis, and TNM stage, while the expression of PD-L1 in exosomes and tissues did not correlate [137]. In contrast, another study demonstrated that the amount of plasma-derived PD-L1+ exosomes was correlated with the level of PD-L1 expression in tumor tissue [138].

The role of plasma-derived exosome miRNAs in patient selection prior to ICI therapy was explored by Peng et al. [139]. They evaluated 30 advanced NSCLC patients who received immunotherapy. Three miRNAs from the hsa-miR-320 family (hsa-miR-320b, hsa-miR-320c, and hsa-miR-320d) were found to be promising predictors, one of which was identified as a possible target for anti-PD-1 therapy (hsa-micro-125b-5p). The upregulation of all three miRNAs was associated with adverse anti-PD-1 responses in patients with progressive disease. Additionally, hsa-miR-125b-5p was down-regulated in patients who responded to anti-PD-1 therapy. Therefore, continuous monitoring of hsa-miR-125b-5p levels during anti-PD-1 therapy is suggested to be an on-treatment diagnostic tool, especially for patients exhibiting delayed response or pseudo-progression. In conclusion, the detection of exosomal miRNAs levels in plasma may more accurately and dynamically reflect the status of tumor cells, and may allow monitoring of tumor progression during treatment.

Besides, the ability of exosomes to carry different cargoes (including drug and molecular information) to the recipient cells enables them to become a new tool for cancer therapy. On the one hand, considering that they contain antigens specific to tumors, exosomes can be used as an effective vaccine against cancer. In a clinical study, it was evaluated about the safety and efficacy of MAGE peptide-loaded exosomes originated from autologous dendritic cells (DEX) of NSCLC patients as a tumor vaccine [140]. The authors observed an immune response with enhanced NK cell activity and a T-cell response targeting MAGE peptides, and long-term disease stabilization achieved in some patients. Another clinical study evaluated the clinical benefit of the oncogenic antigen IFN-γDEX in NSCLC patients with no disease progression post chemotherapy [141]. DEX-induced enhanced anti-tumor capacity of NK cells has already been established in advanced NSCLC patients with NKp30 expression deficiency. These studies illuminate the potential application of exosomes derived from dendritic cells as anti-cancer vaccine. On the other hand, exosomes can be a potential delivery vehicle for biomolecules and drugs. Compared to other drug delivery tools, exosomes have a number of advantages such as small toxicity, low immunogenicity, and the capability of transporting across the blood-brain barrier [142]. Effective loading of docetaxel (DTX) into exosomes by electroporation remarkably improved cellular intake in vitro assessment and demonstrated better targeting to mouse tumor tissue, compared to free DTX [143]. In another study, engineering of targeted tLyp-1 exosomes with high transfection efficiency for lung cancer and cancer stem cells enables to knock down target genes in cancer cells and diminish the stemness of cancer stem cells [144]. Thus, exosomes may provide a prospective gene delivery platform for future treatment.

In summary, exosome-based assessment of PD-L1 status is a promising biomarker for screening NSCLC patients who might benefit from immunotherapy. The first and most important challenge to make the implementation of exosomes in clinical practice feasible is the standardization of exosome isolation and purification methods.

### Other blood markers and immunotherapy of NSCLC

#### Circulating non-coding RNA

Current research on the role of non-coding RNAs in lung cancer immunotherapy has focused on miRNAs. Fan et al. reported that certain circulating miRNAs changes affect response and survival during ICI therapy [145]. A discovery analysis using miRNA profiling was performed by the authors, revealing a different profile of 27 miRNA expressions (22 with high expression and 5 with low expression) between 19 NSCLC patients who responded to immunotherapy and 27 patients who did not. Then in an independent cohort of patients, 10 highly expressed miRNAs were validated. The relevance of this research is that the level of the 10 highly expressed miRNA patterns increased longitudinally from pre- to post-treatment in disease-responsive patients, which correlated with better survival. Another study showed that a miRNA signature classifier (MSC) consisting of 24 miRNAs could distinguish NSCLC patients who benefited from anti-PD-L1 immunotherapy from those who did not benefit [146]. All these results support miRNA as a biomarker to predict immunotherapy response non-invasively in NSCLC patients. In addition, lncRNAs and circRNAs among non-coding RNAs were also found to be potential lung cancer biomarkers. For example, lncRNAs XIST and HIF1A-AS1 were significantly increased in the serum of NSCLC patients [147]. Another study identified a set of circRNAs with prognostic roles in NSCLC, including two overexpressed (circ-0005962 and circ-0003958) and low-expressed (has-circ-0086414 and has-circ-0001936) circRNAs [148]. However, several limitations must be considered, such as the lack of consensus on isolation methods, the heterogeneity of studies, and the small cohorts of patients used. In summary, circulating ncRNAs are very promising as appropriate biomarkers for clinical disease management [149, 150].

#### T-cell receptor repertoire

T-cell receptor (TCR) is a characteristic marker on the surface of T cells that serves to recognize antigens. TCR sequences contain a complementary determining-region 3 (CDR3) which ensures antigen specificity and immunological response strength [151]. Each T-cell has a unique TCR sequence, and the TCR repertoire of an individual is composed of the different TCR sequences, their distribution, and frequency. Variations in TCR repertoire can be detected by high-throughput sequencing and have been investigated in multiple types of cancer, including NSCLC. A study by Han et al. focused on the TCR repertoire of TCD8+PD-1+ cells in 40 patients with NSCLC treated with anti-PD-1 or anti-PD-L1 therapy [152]. They observed that patients with high TCR variability had significantly higher mean PFS before and after treatment than those with lower TCR variability (before treatment: 6.4 months vs. 2.5 months; 4–6 weeks after first treatment: 7.2 months vs. 2.6 months). In addition, CX3C chemokine receptor 1 (CX3CR1), a marker of T-cell differentiation, was investigated for its role in predicting immunotherapeutic responses by Yamuchi et al. Peripheral blood mononuclear cell (PBMC) samples from 36 NSCLC patients treated with pembrolizumab or nivolumab were analyzed, and researchers found that positive clinical outcomes were correlated with increased frequency of the CX3CR1-positive CD8+ T cells in circulating blood [153]. In conclusion, the diversity and clonality of TCR can be used as a biomarker to monitor the response to immunotherapy in patients with NSCLC [152]. However, the challenge of high false-positive rates due to aggregation of functionally distinct clones or the presence of artificial clones needs to be overcome before the implementation in clinic is feasible.

#### Circulating immune cells

The role of blood cell populations, including key immune cell subsets, as circulating markers of prognosis at diagnosis and baseline in cancer patients has been historically studied. Lymphocytes play a core role in immunotherapy-induced anti-tumor responses. In a study that included 34 lung cancer patients, including 28 with NSCLC and 6 with SCLC, researchers conducted a comprehensive analysis of several immune populations and found that higher natural killer (NK) cells levels and CD4+/CD8+ cell ratios predicted prolonged PFS at baseline with ICI therapy. NSCLC patients with lower levels of regulatory T cells (Tregs) at baseline achieved better outcomes, regardless of ICI or ICI combined therapy [154]. In addition, several studies have linked neutrophil-to-lymphocyte ratio (NLR) to OS, PFS, and ORR in advanced NSCLC treated with immunotherapy [155–157]. NLR and platelet-to-lymphocyte ratio (PLR) have also been found to be predictors of irAEs in patients with advanced NSCLC treated with ICIs [158]. Besides, platelets play an important role in systemic and local anti-cancer responses. They comprise a broad library of RNA species that provide biomolecules for diagnosis and prognosis, prediction or subsequent biomarkers [159]. At last, routine complete blood count (CBC) tests in combination with other biomarkers has been shown to guide ICI-based treatment decisions in NSCLC patients. For example, combined NLR and hemoglobin (HGB) can predict response of ICI, and patients who show high NLR and low HGB before treatment have worse OS [160]. Although metrics like PLR and NLR obtained from routine blood analysis provide interesting opportunities for monitoring treatment outcomes, circulating immune cells are still lacking well-designed studies that demonstrate their true potential as biomarkers.

#### Peripheral blood cytokine

Cytokines are playing a key role in the inflammatory tumor microenvironment that is closely associated with cancer progression. They transmit information between cells, facilitate the recruitment of immune cells into the tumor microenvironment and also affect the expression of immune checkpoint receptors and ligands, thus influencing ICI responses. In the study by Boutsikou et al., 26 patients with NSCLC were treated with ICI. IFN-γ, tumor necrosis factor-α (TNF-α) and a group of ILs were measured in patients’ peripheral blood to assess their prognostic and predictive role. Analyzing these cytokines by flow cytometry at diagnosis and 3 months after starting immunotherapy, they found that patients with higher levels of IFN-γ, TNF-α, IL-1β, IL-2, IL-4, IL-5, IL-6, IL-8, IL-10 and IL-12 demonstrated improved ICI response and prolonged survival than those with lower levels [161]. There are also studies focusing on specific cytokines, and the results all support that circulating cytokine levels have the potential to reflect the state of the tumor immune microenvironment and the clinical outcomes of ICI therapy, particularly IL-6 and INFγ levels [162–164]. However, there are some limitations to the current research, such as the small amounts of patients involved in studies, the different treatment approaches, and the lack of clear boundaries. Validating the potential of circulating cytokines and translating them into clinical application require further research.

### Gut microbiota and immunotherapy of NSCLC

Feces-based gut microbiota have been discovered to be involved in the development, progression and metastasis of various types of cancers in the intestinal and extraintestinal tissues [165, 166]. In addition, gut microbes can be involved in the induction of inflammation or indirectly in cancer therapy through immunosuppression, ultimately affecting the outcome of anti-tumor therapy [167]. Recent preclinical and clinical studies have linked gut microbiota composition to specific and toxic responses to ICIs [168, 169]. The research of microbiota is still in its infancy, but it holds great promise for monitoring immunotherapy patients with NSCLC.

## Conclusion and future prospects

As a non-invasive method of obtaining biological samples, liquid biopsy is growing in importance in cancer treatment. Samples from the tumor microenvironment and tumor tissue may provide valuable diagnostic, prognostic, and therapeutic response information. This sample collection technique is particularly important in NSCLC cases, since tissue biopsies are highly invasive and usually have a high intra-tumor heterogeneity bias. Unlike tissue biopsy, liquid biopsy biomarkers are more readily available and can be identified numerous times during treatment. Its emergence is an important step toward easier diagnosis and disease management.

Due to the presence of hopeful results from observational research and the continuous development of analytical platforms, liquid biopsy diagnostics based on cfDNA has entered the next stage of development. The findings of somatic mutations in cfDNA associated with genes sensitive or resistant to immunotherapy will help physicians make better treatment decisions, but the isolation and analysis of cfDNA remains challenging. There are multiple sources of plasma cfDNA, such as fetal and germline, as well as cfDNA mutations of hematopoietic origin, which may be mistaken for mutations of tumor origin [170]. Therefore, the detection results of cfDNA are prone to false positive. In addition, the sensitivity and specificity of ctDNA detection are also affected by ctDNA variation. The variation of ctDNA may change with the evolution of tumor and be affected by different treatments. It has been suggested that plasma levels of ctDNA are a sign of treatment response, as a reduction in ctDNA is linked to with improved outcomes in patients receiving ICI treatment. However, there is no clear consensus on the method to measure the overall levels.

CTCs, as intact living cells with tumor-specific information, can also be used for diagnosis and therapeutic assessment of cancers. However, the identification and isolation techniques of CTC lag far behind those of ctDNA. This raises evident problems in NSCLC, as the number of CTCs detected in the blood of NSCLC patients is consistently lower than in other cancer types. This may be due, at least in part, to the fact that CTC isolation techniques rely heavily on EpCAM-dependent approaches that can only identify epithelial cells. New markers are needed to detect tumor cells that undergo epithelial-mesenchymal transition (EMT). In addition, single-cell analysis or cluster analyses are possible because of methods capable of preserving and capturing intact CTCs. Characterization of CTCs in single or clusters would enable a more accurate assessment of each patient’s entire cancer.

Analysis of extracellular vesicles may be able to serve as a less invasive alternative to traditional analysis of PD-L1 expression in primary tumors. However, EVs are of complex origin and particular markers are required to reliably distinguish cells of tumor origin and thus identify EVs of tumor origin. The clinical application of circulating ncRNAs as a non-invasive predictive biomarker still needs to be validated by numerous studies. TCR determinations have also shown great promise in response biomarker settings, and analysis of T-cell clonality may reveal the extent of tumor antigen-driven T-cell expansion and contribute to understanding the mechanisms of T-cell tolerance to cancer antigens. Moreover, the status of TME plays a major role in ICIs response, and thus the counts of distinct circulating immune cell populations in TME are also relevant to the immunotherapeutic response. Similarly, cytokines in TME can also suggest the inflammatory state of the tumor and surrounding TME, and significant results have been obtained regarding the correlation of IL-6 and INF-γ levels with immunotherapy efficacy.

Currently, there is still much work to be done. Most of these liquid biopsy methods are in the ongoing research phase, and large prospective clinical trials must be performed to provide evidence of their clinical utility. In addition, body fluids other than plasma may sometimes be more useful in NSCLC and therefore their full potential should be explored. Moreover, the predictive role of many biomarkers is not absolute due to the complexity of the immune microenvironment, the interaction of multiple factors, and the limitations of current knowledge. In clinical practice, a single index should not be used as an evaluation criterion. Liquid biopsy data should be combined with clinical indicators such as tissue biopsy, imaging findings, and tumor markers to comprehensively monitor and guide the clinical treatment of NSCLC patients in real time and provide more accurate information about the actual conditions of patients.

In summary, liquid biopsy offers unprecedented possibilities for exploring biomarkers of response to immunotherapy. Although larger study cohorts and independent verifications are needed before clinical practice, its potential value in predicting the immune response in patients with NSCLC is beyond doubt. It is hoped that in the near future, as technological means advance, new biomarkers continue to be discovered and more proven multi-omic, multi-parametric predictive models are developed to help clinicians achieve greater optimization and precision in immunotherapy prediction and protocol development.

## Data Availability

The data used and analyzed are available from the corresponding author upon reasonable request.
